# Supplementary material for: Stacking Model‐Based Classifiers for Dealing With Multiple Sets of Noisy Labels
Source: Biom J. 2025 Mar 12;67(2):e70042. doi: 10.1002/bimj.70042 (PMC11898607; doi:10.1002/bimj.70042)
Supplement: Supplementary file 1 — Supporting Information [file BIMJ-67-e70042-s001.zip › density-based-ensemble-model-main/competitors_real_data/netlab-master/netlab-master/help/gppak.htm]

Netlab Reference Manual gppak


# gppak

## Purpose

Combines GP hyperparameters into one vector.

## Synopsis

```
hp = gppak(net)
```

## Description

`hp = gppak(net)` takes a Gaussian Process data structure `net` and
combines the hyperparameters into a single row vector `hp`.

## See Also

`gp`, `gpunpak`, `gpfwd`, `gperr`, `gpgrad`

---

**Pages:**
Index


---

Copyright (c) Ian T Nabney (1996-9)
